# DevMouse, the mouse developmental methylome database and analysis tools

**DOI:** 10.1093/database/bat084

**Published:** 2014-01-09

**Authors:** Hongbo Liu, Rangfei Zhu, Jie Lv, Hongjuan He, Lin Yang, Zhijun Huang, Jianzhong Su, Yan Zhang, Shihuan Yu, Qiong Wu

**Affiliations:** ^1^Department of Developmental Biology, School of Life Science and Technology, State Key Laboratory of Urban Water Resource and Environment, Harbin Institute of Technology, Harbin 150001, China, ^2^Department of Computational Systems Biology, College of Bioinformatics Science and Technology, Harbin Medical University, Harbin 150081, China, ^3^Department of Food Science, School of Food Science and Engineering, Harbin Institute of Technology, Harbin 150001, China and ^4^Department of Respiratory Medicine, the First Affiliated Hospital of Harbin Medical University, Harbin 150001, China

## Abstract

DNA methylation undergoes dynamic changes during mouse development and plays crucial roles in embryogenesis, cell-lineage determination and genomic imprinting. Bisulfite sequencing enables profiling of mouse developmental methylomes on an unprecedented scale; however, integrating and mining these data are challenges for experimental biologists. Therefore, we developed DevMouse, which focuses on the efficient storage of DNA methylomes in temporal order and quantitative analysis of methylation dynamics during mouse development. The latest release of DevMouse incorporates 32 normalized and temporally ordered methylomes across 15 developmental stages and related genome information. A flexible query engine is developed for acquisition of methylation profiles for genes, microRNAs, long non-coding RNAs and genomic intervals of interest across selected developmental stages. To facilitate in-depth mining of these profiles, DevMouse offers online analysis tools for the quantification of methylation variation, identification of differentially methylated genes, hierarchical clustering, gene function annotation and enrichment. Moreover, a configurable MethyBrowser is provided to view the base-resolution methylomes under a genomic context. In brief, DevMouse hosts comprehensive mouse developmental methylome data and provides online tools to explore the relationships of DNA methylation and development.

**Database URL**: http://www.devmouse.org/

## Introduction

DNA methylation is an epigenetic modification involving the addition of a methyl group at the 5′-position cytosine in DNA sequence (1). In mammalian genomes, most cytosines in CpG dinucleotides are methylated by DNA methyltransferases, whereas those in CpG islands are protected from methylation (2). DNA methylation plays crucial roles in transcriptional regulation, genomic imprinting, X chromosome inactivation and long-term repression (3,4). It has been reported that DNA methylation is highly dynamic during mammalian development and participates in regulation of embryogenesis, cell-lineage determination and the genesis of germ cells (5,6). The aberrant DNA methylation programming and reprogramming during development would cause the inheritance of epigenetic mutations that have been found to be associated with human diseases as suggested by studies on mouse models (7,8). Owing to the important regulatory roles and the improvement of high-throughput technologies, DNA methylation is extensively studied in the development progress of the mouse, which is the model organism most closely related to humans. Studying the influence of DNA methylation on developmental genes in mouse development should offer insight into the mechanisms affecting mammalian development and human developmental disorders, given the ethical issues of human studies.

Using bisulfite conversion coupled with second-generation sequencing, researchers from around the world have profiled DNA methylomes for different developmental stages of the mouse and analyzed the DNA methylation during mouse development (9–16). By reduced representation bisulfite sequencing, Meissner *et al.* mapped the genome-scale DNA methylation maps of pluripotent and differentiated cells, and found DNA methylation undergoes demethylation and then remethylation during the development of the mouse nervous system (9). By profiling and studying a genome-scale base-resolution timeline of DNA methylation in the pre-specified embryo, Smith and colleagues found DNA methylation dynamics in the early mammalian embryo (13). A base-resolution allele-specific DNA methylation map in the mouse genome revealed the roles of differential methylation in the regulation of imprinting and allele-specific gene expression in mammalian cells (14). Most recently, Kobayashi *et al.* profiled the high-resolution DNA methylomes of primordial germ cells via whole-genome shotgun bisulfite sequencing and found gender-specific reprogramming from E10.5 to E16.5 (15). The integration and depth mining of these methylomes in temporal order should help us to gain further knowledge about dynamic DNA methylation during development from a global perspective. It should be possible to discover potentially novel developmental genes/regions regulated by DNA methylation via integrating methylomes across multiple developmental stages and identifying differentially methylated genes during development. Integrative analysis of methylomes scattered in different data recourses is a great idea, but costly and difficult to implement for experimental biologists with limited bioinformatics experience.

Currently, there are a few databases involved in DNA methylation. The data resource databases National Center for Biotechnology Information (NCBI) Epigenomics (17), NGSmethDB (18) and MethDB (19) were designed as a great data pool for epigenetic modification data or DNA methylation data stored according to the experiments and samples. NCBI Epigenomics provides genome-wide maps of DNA and histone modifications from a diverse collection of epigenomic data sets. NGSmethDB is a database for storage and retrieval of methylation data by next-generation sequencing. MethDB focuses on environmental effects on DNA methylation. The disease methylation databases MethyCancer (20), DiseaseMeth (21) and MethylomeDB (22) were developed to study the aberrant DNA methylation alterations in human disorders. MethyCancer focuses on the integrated cancer-related DNA methylation data. DiseaseMeth is a web-based resource focused on the aberrant methylomes of human diseases, and MethylomeDB is a database containing genome-wide DNA methylation profiles for neurodevelopmental and neuropsychiatric disorders. However, there is no specialized and comprehensive database that focuses on storage of mouse developmental methylomes in temporal order or provides convenient analysis tools for in-depth mining of methylation dynamics from these data.

Thus, DevMouse was developed to store the mouse developmental methylomes in temporal order and provide online analysis tools for mining of the developmental genes/regions with dynamic DNA methylation during mouse development. The current version of DevMouse stores temporally ordered DNA methylomes covering multiple developmental stages, which should be useful for a wide range of developmental biologists. DevMouse supports users to search for the methylation patterns of various genome items such as genes, microRNAs (miRNAs), long non-coding RNAs (lncRNAs), CpG islands and other genome regions, which should benefit broad researchers focusing on molecular biology from genes to specific genome regions. Furthermore, the convenient analysis tools are provided for depth mining of novel knowledge from integrated methylomes in a global perspective. DevMouse also includes a configurable methylation browser, MethyBrowser, by which base-resolution developmental methylomes can be shown under a mouse genome context. All the search and analysis results can be viewed as graphs for view and downloaded as figures or tables for further analysis.

## Database construction and content

**Figure 1.**
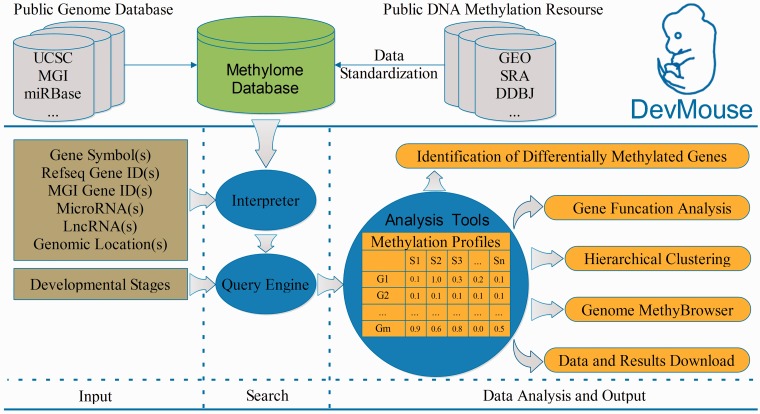
Overview of building and workflow of DevMouse. DevMouse integrates DNA methylomes of mouse development from public DNA methylation resources and the mouse genome information. Users can input multiple genome items to the query engine to gain the methylation profiles of genes/regions in different developmental stages. The analysis tools enable users to carry out online analysis on the methylation profiles, including identification of differentially methylated genes/regions, gene function analysis, hierarchical clustering and visualization in MethyBrowser. All search and analysis results can be downloaded as flat format for further analysis.

DevMouse was designed to store high-throughput DNA methylation data during mouse development in temporal order. The current version of DevMouse consists of 32 DNA methylomes in single-base resolution across 15 mouse developmental stages that were collected from public DNA methylation resources (23–28) and genome information (genes, mRNAs, lncRNA, CpG islands and others) obtained from public genome databases (29–33) (Figure 1 and Table 1). These methylomes are profiled by the next-generation sequencing technologies coupled with bisulfite conversion. In these methylomes, methylated cytosine can be distinguished from unmethylated cytosine by the presence of a cytosine versus thymine residue during sequencing. The proportion of methylated cytosine is treated as the methylation level, which ranges from 0% representing unmethylated cytosine to 100% fully methylated cytosine. All methylation data were subsequently processed according to the same procedure. Before being finally stored, the methylomes from assemblies other than the University of California, Santa Cruz (UCSC) July 2007 mouse reference sequence (mm9, NCBI build 37) were converted into mm9 by the LiftOver tool from UCSC (30). All data available can be downloaded from the download page, which lists detailed information about the data including experiment name, experimental technology, cell/tissue type, developmental stage, sex, author information, download links and external database links. Based on these high-throughput cytosine methylomes, DevMouse provides the basic operations, search, analysis, view and download (Figure 1). A flexible query engine is provided for acquisition and investigation of the methylation profiles of genes/regions of interest. Powerful analysis tools written in Java facilitate in-depth mining of novel knowledge about DNA methylation and development. Moreover, the methylation information and novel findings can be viewed by the visualization modules based on Apache Batik Scalable Vector Graphics (SVG) toolkit, and can be downloaded before exiting the browser for further reuse.

**Table 1.** DevMouse data content and statistics as of 1 September 2013

Data content	Data statistics
DNA methylation	
Single base DNA methylome	32
Profiled by RRBS	26
Profiled by BS-Seq/WGSBS	6
Developmental stage	15
Genome information	
Cytosine	1068446432
With methylation data	42685552
Gene symbol	23417
With methylation data	23321
RefSeq gene	31263
With methylation data	31094
MGI gene	62083
With methylation data	52448
MicroRNA	3212
With methylation data	1478
LncRNA	1335
With methylation data	1335
CpG island	16026
With methylation data	15991
Other integrated data	
Gene expression	31840
Transcription factor binding site	13483
Repeat	4881442
Single-nucleotide polymorphism	14893502
Class of function annotation	11

## Database use and access

### Search methylation profiles of genes across developmental stages

**Figure 2.**
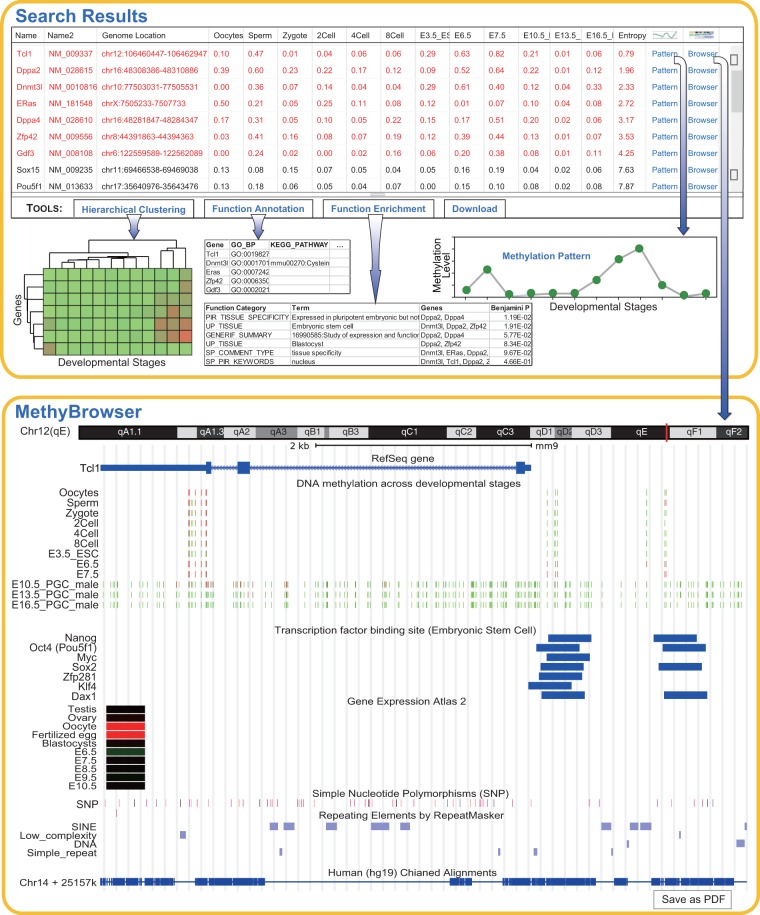
Search and analysis results on 19 genes during sperm development. Shown at the top is the default table and view generated by the query and analysis tools. The table shows the methylation profiles of 19 genes across 13 developmental stages and the sorted entropy values representing the methylation variation. The genes in red are differentially methylated across multiple stages. Using the buttons in the toolbar, the user can easily carry out online analysis including hierarchical clustering, gene function annotation and enrichment. The analysis results are shown as images or tables. The methylation pattern can be shown in a pop-up panel after clicking ‘Pattern’ link. When the ‘Browser’ link is clicked, the gene-centric methylation profile of gene *Tcl1* and related genomic information are viewed in MethyBrowser as shown at the bottom.

The search toolkits in the homepage of DevMouse can be used to acquire the methylation states for any given genes/regions of interest across multiple developmental stages. In the current version, DevMouse supports six kinds of gene IDs including the official gene symbol, Refseq gene, MGI gene, mRNA and lncRNA. For a gene >500 bp, the methylation state is calculated as the mean methylation level of the cytosines located in the promoter region from upstream 2 kb to downstream 500 bp of the transcriptional start site, and the methylation state of a gene <500 bp is calculated as the mean methylation level of the cytosines located in the whole gene region. As an example, we imported 19 gene symbols (listed in gene example 1 in the homepage) that were used for induction of pluripotent stem cells (34) and selected 13 developmental stages during mouse sperm development (stage example 1 in the homepage). The search result is displayed by default as an overview table that summarizes the methylation profiles of genes across multiple developmental stages as well as gene information and chromosomal location (Figure 2). The table contains links to a methylation pattern panel in which the methylation profile of a selected gene can be viewed, and links to the MethyBrowser in which the user can view the methylation profile as well as genomic information, according to the specified view parameters. The whole query result data can be downloaded to local computers from the download links in the overview panel.

### Analysis tools to explore methylation dynamics and gene function

DNA methylation is highly dynamic during mouse development, and hypermethylation of gene promoter inhibits a few genes including developmental genes at a specific developmental stage (35,36). However, few genes were documented to exhibit differential promoter methylation during a whole development process due to less availability of DNA methylation data. As bisulfite sequencing enables profiling of mouse developmental methylomes on an unprecedented scale, experimental biologists with limited bioinformatics experience challenges in integrating and mining these data. To facilitate integrative analysis of methylomes, DevMouse offers online tools for quantitative analysis: (i) entropy-based quantification of methylation variation, (ii) identification of differentially methylated genes/regions, (iii) hierarchical clustering analysis of methylation profiles and (iv) gene function annotation and enrichment (Figure 2). An entropy-based approach is integrated to quantify methylation variation of a gene across multiple developmental stages, with lower entropy indicating greater methylation variation as described in our previous study (37). The genes with entropy lower than a threshold are identified as differentially methylated genes that are marked in red in the result table. Furthermore, the stage specificity of a differentially methylated gene in each stage is measured by an algorithm embedded in DevMouse. The hierarchical clustering analysis tool can be used to study the methylation similarity among genes or stages, and identify the genes with similar methylation patterns across developmental stages. The functional similarity in 11 kinds of functions for a differentially methylated gene set can be retrieved directly by the tools for gene annotation and enrichment, which is based on DAVID API (38). Using these analysis tools, we identified differentially methylated genes across multiple stages in each of the development lines stored in the current database, and performed clustering of methylation profiles for differentially methylated genes. These differentially methylated genes and clustering maps can be obtained from the DMGenes page. One merit of these tools is that they are highly automatic for analyzing given genes, facilitating specific analysis focusing on identification of a functional gene set such as potentially novel developmental genes. All analysis results by these tools can be downloaded as in the figures or tables for further analysis.

### MethyBrowser

Moreover, a configurable methylation browser, MethyBrowser, is developed using the Apache Batik SVG toolkit for users to view the methylomes and genome information simultaneously (Figure 2). The genome MethyBrowser connecting to a MySQL backend is used to show the methylation profiles of imported gene/region across multiple developmental stages at single-base precision. A color gradient from green (methylation value = 0%) to red (methylation value = 100%) is used to display the numeric methylation states of the cytosines. MethyBrowser also visualizes other available genomic annotations including chromosomal location, base sequence, gene structure, lncRNA, miRNA, CpG island, transcription factor binding site, gene expression, single-nucleotide polymorphism, repeat elements, sequence tagged site along the mouse draft assembly and the alignment in human genome (hg19) of this region. It should be noted that base sequence is shown only when the region is <150 bp. The alignment in the human genome (hg19) may be helpful for exploring the potential roles of orthologous genes/regions in the human genome. Features of MethyBrowser include the ability to view the region by specifying the genomic coordinates, to zoom and move the given region, to show and hide certain feature tracks by mouse-click configuring, as well as to access the function of the genes by links to Entrez Gene in NCBI (39). As shown in Figure 2, links to MethyBrowser are also available in the search result page. By the ‘Save as PDF’ button, the browser graphic can be saved as PDF images that can be printed with Acrobat Reader and edited by many drawing programs such as Adobe Illustrator.

## System design and implementation

DevMouse was constructed based on three major software components: an Apache Tomcat web server, a MySQL relational database and Java-based computational services. The backstage processing programs were written in Java, which are available on request. The web services were developed using Apache Struts2, a Java web application framework, and iBATIS, a persistence framework that automates the mapping between MySQL databases and objects in Java, both of which help guarantee the high performance and stability of the web services. Browser-based interfaces were built in JSP and AJAX. The Apache Batik SVG toolkit was used to render, generate and manipulate the SVG dynamically. DevMouse allows users to access all of the key features of the web application through their mobile device. DevMouse is available at http://www.devmouse.org.

## Future perspective

The current version of DevMouse is the first release of our database. Although it contains a wealth of development-specific DNA methylomes in the mouse, which will be of great use both for experimental and bioinformatics researchers, the available data and functionality are still limited. Aiming to build a DNA methylome database focusing on the mouse development, continued efforts will be made to update the DevMouse data, add more methylation analysis tools and improve the functionality of the database and MethyBrowser. As the rapid profiling of DNA methylomes in more and more samples based on high-throughput bisulfite sequencing, we will continuously collect the latest data sets in different developmental stages of the mouse to keep DevMouse up-to-date. We would like to invite and encourage the scientific community to submit their methylation data about mouse development to keep DevMouse updated and comprehensive. As a resource to study the potential roles of DNA methylation in mouse development, DevMouse could be extended with utilities for the identification and confirmation of developmental markers related to DNA methylation from large-scale methylome data (40). The MethyBrowser will be improved to display strand-specific methylation in higher resolution and be extended by more configurable functionalities. Because chromatin modifications including histone modification have also been reported as dynamic marks of mouse developmental genes, we would extend the research scope and integrate chromatin modification data into DevMouse. We hope our continuous efforts working on the database will contribute to the understanding of epigenetic regulation in mouse development and modeling human development and disease.
